# Biologic Drug Prices in Medicare Part B After Entry of Biosimilars to the Market

**DOI:** 10.1001/jamanetworkopen.2025.42937

**Published:** 2025-11-11

**Authors:** Abdullah Abdelaziz, Aaron N. Winn, Stacie B. Dusetzina, Aaron P. Mitchell

**Affiliations:** 1 Department of Pharmacy Systems, Outcomes and Policy, College of Pharmacy, University of Illinois Chicago, Chicago; 2 Department of Health Policy, Vanderbilt University Medical Center, Nashville, Tennessee; 3 Vanderbilt Ingram Cancer Center, Nashville, Tennessee; 4 Department of Epidemiology and Biostatistics, Memorial Sloan Kettering Cancer Center, New York, New York

## Abstract

**Importance:**

Price reductions for originator biologics with biosimilar competitors have been modest in the US. This may be due to the US reimbursement model, where clinicians or their employing medical institution receive payment based on the percentage of the cost billed, incentivizing the highest-cost product. Currently, US policymakers are debating changing this policy. However, a clear understanding of price reductions achieved by the current system is needed to inform future reform efforts.

**Objective:**

To assess the associations of biosimilar entry with the prices of originator biologics under Medicare Part B reimbursement.

**Design, Setting, and Participants:**

This cohort study used bayesian structural time series models to analyze Medicare Part B Drug average sales price (ASP) data from quarter 1 of 2005 to quarter 1 of 2025, including 7 biologics with biosimilars available for at least 3 years (bevacizumab, epoetin, filgrastim, infliximab, pegfilgrastim, rituximab, and trastuzumab).

**Exposure:**

Biosimilar entry.

**Main Outcomes and Measures:**

Relative differences in observed ASPs for originators and biosimilars vs the bayesian structural time series–estimated counterfactual ASPs for originators had biosimilars not entered the market.

**Results:**

Analysis included 7 originator biologics that entered the market between 1989 and 2024. The number of biosimilars marketed during the study period ranged between 1 and 6 for originator biologics studied. Biosimilar entry was associated with changes in the ASP of the originator biologic of −7.4% (95% prediction interval, –10.5% to −4.3%) at 1 year, −31.7% (95% prediction interval, –41.6% to −21.9%) at 3 years, and −43.1% (95% prediction interval, –61.3% to −24.8%) at 5 years. Savings varied by drug, with the largest price reductions observed with pegfilgrastim and infliximab, with 5-year price reductions of −82.0% (95% prediction interval, –95.2% to −54.0%) for pegfilgrastim and −62.3% (95% prediction interval, –73.4% to −45.2%) for infliximab. Biosimilar prices were generally lower than the originator biologics at all postentry periods and decreased over time.

**Conclusions and Relevance:**

In this cohort study, counterfactual analysis found that price reductions from biosimilar entry were more substantial than suggested by simpler pre-post comparisons. However, these savings remained less than those achieved by small-molecule generics, highlighting the need for policy reforms to enhance market competition and realize greater savings.

## Key Points

**Question:**

How are the prices of originator biologic drugs reimbursed under Medicare Part B associated with the entry of biosimilar drugs to the market?

**Findings:**

This cohort study (N = 7 drugs) used bayesian structural time series models to construct a synthetic control, estimating what originator drug prices would have been without biosimilar competition (the counterfactual). Compared with this counterfactual, observed prices decreased overall, with substantial reductions for pegfilgrastim and infliximab.

**Meaning:**

This study suggests that biosimilar entry to the market was associated with modest price reductions, but policy changes are needed to enhance the cost-saving potential under Medicare Part B.

## Teaser

This cohort study evaluates the associations of biosimilar entry to the market with the prices of originator biologics under Medicare Part B reimbursement.

## Introduction

Biologic drugs, used for treating complex and chronic conditions, such as cancer and autoimmune disorders, have significantly advanced patient care, but at substantial financial cost.^1,2^ In the US, biologics account for nearly 46% of prescription drug spending despite representing a small fraction of use.^2^ These high costs can be attributed to intricate manufacturing processes, extended patent protections, and limited market competition, making biologics one of the primary factors associated with escalating health care expenditures.^3^ In 2009, the Biologics Price Competition and Innovation Act created a regulatory pathway for biosimilars—highly similar alternatives to reference biologics—with the goal of lowering spending for these products after their patents and exclusivity periods had expired.^4^ Although biosimilars have shown potential for significant cost savings, their impact in the US market has been considerably less pronounced compared with Europe and other regions, where broader acceptance and uptake of biosimilars have led to greater reductions in drug spending.^5,6^

Multiple market, regulatory, and behavioral factors have influenced the slow uptake of biosimilars in the US. A key barrier is the Medicare Part B reimbursement structure, in which biosimilar drugs are paid based on the average sales price (ASP) plus an add-on fee that is a percentage of the referent biologic drug’s price, making lower-cost biosimilars no more profitable to hospitals and clinicians than higher-priced biologics.^7,8,9^ This reimbursement model contrasts with capitated models such as Medicare Advantage and managed care settings, wherein financial incentives to reduce expenditures may expedite the adoption of biosimilars.^10,11,12^ In addition, financial structures within programs such as the 340B Drug Pricing Program can sometimes favor originator biologics over biosimilars, even when biosimilars are less expensive, further complicating uptake.^13^ Despite targeted efforts, such as implementing specific financial incentives to boost biosimilar use,^14,15^ recent studies suggest that overall adoption remains modest due to strategic pricing by manufacturers^3^ and limited out-of-pocket savings for patients, reducing the incentive to switch to a lower-cost biosimilar.^16^ Moreover, prescriber hesitancy and aggressive marketing by originator manufacturers contribute to the slower-than-expected adoption of biosimilars.^17,18^

Prior evaluations of the association of biosimilar prices with the prices of originator biologics have typically compared postentry prices with those immediately preceding market entry.^19,20,21^ This pre-post comparison likely underestimates the true association between prices, as it fails to account for underlying price trends and the likelihood that originator biologic prices would have continued to increase without new competition.^2^ To address this limitation, we used a synthetic control approach to construct a robust counterfactual for each originator biologic.^22,23^ By modeling the counterfactual price trajectory without biosimilar competition, we aimed to provide a more accurate and comprehensive estimate of the price reductions directly associated with biosimilar competition.^24,25,26^

## Methods

### Data Source

We used the publicly available Medicare Part B Drug ASP Pricing Files for quarter 1 of 2005 through quarter 1 of 2025.^27^ The datasets are curated via a structured process involving manufacturers’ reporting, and calculation and validation by the Centers for Medicare & Medicaid Services (CMS). Manufacturers report the ASP of their drugs each quarter, which is calculated from actual sales data, net of rebates. The manufacturers submit these data to CMS, validating it for accuracy and completeness. This study did not constitute human participants research, and, per the Common Rule, institutional review board approval and informed consent were not required. We followed the Strengthening the Reporting of Observational Studies in Epidemiology (STROBE) statement for cohort studies.^28^

To ensure our analysis used the true ASP, we back-calculated it from the Medicare payment allowances. This required accounting for a policy change that occurred in quarter 4 of 2022. For quarters before this date, we reversed the standard ASP + 6% payment rule. From quarter 4 of 2022 onward, we determined the true ASP by reversing the temporary ASP + 8% rule for qualifying biosimilars and the standard ASP + 6% rule for all other products.^29^

We selected a set of biologic agents that had marketed biosimilar competitors for at least 3 calendar years to enable a reliable estimation of the association of biosimilar market entry with the price of originator biologics. Specifically, we searched the US Food and Drug Administration (FDA) Purple Book Database of Licensed Biological Products^30^ for all biologic products with approved biosimilar competitors. We included all originator biologics that are reimbursed under Medicare Part B and had 1 or more biosimilar competitors marketed in 2021 or earlier: bevacizumab, epoetin, filgrastim, infliximab, pegfilgrastim, rituximab, and trastuzumab (Table 1). Due to their different reimbursement rates, we separated epoetin into end-stage kidney disease (ESKD) and epoetin for non-ESKD.

**Table 1.** Biologics Selected for Analysis

Drug	Brand name	Common indications	Market entry date	Biosimilar: market entry date
Bevacizumab	Avastin	Lung cancer, colon cancer	2004 Q1	Mvasi: 2019 Q3 Zirabev: 2019 Q4 Alymsys: 2022 Q4 Vegzelma: 2023 Q2
Epoetin	Epogen	Anemia due to CKD, anemia due to chemotherapy	1989 Q2	Retacrit: 2018 Q4
Filgrastim	Neupogen	Chemotherapy-induced neutropenia, severe chronic neutropenia, bone marrow transplantation	1991 Q1	Granix: 2013 Q4^a^ Zarxio: 2015 Q3 Nivestym: 2018 Q4 Releuko: 2022 Q4
Infliximab	Remicade	Inflammatory bowel disease, rheumatoid arthritis	1998 Q3	Inflectra: 2016 Q4 Renflexis: 2017 Q3 Avsola: 2020 Q3
Pegfilgrastim	Neulasta	Chemotherapy-induced neutropenia	2002 Q1	Fulphila: 2018 Q3 Udenyca: 2019 Q1 Ziextenzo: 2019 Q4 Nyvepria: 2021 Q1 Stimufend: 2023 Q1 Fylnetra: 2023 Q2
Rituximab	Rituxan	Non-Hodgkin lymphoma, chronic lymphocytic leukemia, immune thrombocytopenia purpura, rheumatoid arthritis	1997 Q4	Truxima: 2019 Q4 Ruxience: 2020 Q1 Riabni: 2021 Q1
Trastuzumab	Herceptin	Breast cancer	1998 Q3	Kanjinti: 2019 Q3 Ogivri: 2019 Q4 Trazimera: 2020 Q1 Herzuma: 2020 Q1 Ontruzant: 2020 Q2

Abbreviations: CKD, chronic kidney disease; Q, quarter.

^a^ Tbo-filgrastim (Granix) is not technically a biosimilar, as it was approved via a standalone Biologics License Application before the formal biosimilar pathway existed.

### Statistical Analysis

All analyses were done using R, version 4.4.2 (R Project for Statistical Computing).^31^ For each drug, we assessed the ASP in terms of the number of units that would constitute a single therapeutic dose (without respect to dosing frequency), assuming a patient weight of 70 kg and a body surface area of 1.7 m^2^. We subsequently adjusted these values for inflation to quarter 1 of 2025 US dollars using the Consumer Price Index for All Urban Consumers: Medical Care in the US City Average.^32^

Our primary aim was to estimate the difference between the originator biologic’s observed ASP and its anticipated (counterfactual) ASP in the absence of biosimilar competition (Table 1). To estimate the counterfactual originator biologics’ ASPs, we used the CausalImpact R package^23^ to develop a series of bayesian structural time series (BSTS) models, which use information from control time series data that closely resembles biologics’ ASP trends before biosimilars’ market entry. A BSTS model is a state-space model that forecasts a target time series using a combination of its own local trend and a regression component using contemporaneous control time series. The model constructs a synthetic control by forming a weighted combination of the most predictive biologics from the control pool using a spike-and-slab prior. The spike-and-slab prior selects controls based on how well their prices collectively predict the preintervention trends and dynamics of the target drug’s ASP, not by simply matching absolute price levels at a single point in time.

We identified a set of control biologic drugs for each drug in our sample to estimate its counterfactual price trend in the absence of biosimilar competition. Our goal in selecting potential control drugs was to select drugs in the middle of their patent life cycle, avoiding potential fluctuations or corrections in the first years after market entry or anticipatory changes before biosimilar competition. To do so, we first identified all biologics that (1) received initial FDA approval by January 2015, (2) had no marketed biosimilar competitor as of year-end 2023, and (3) were among the 50 highest-selling drugs in 2022. Because the biologics in our sample entered the market at different time points, each biologic had its own set of controls. We assumed that the control biologics’ price trends were unlikely to be associated with the introduction of biosimilars for different biologic drugs (eg, that market entry of biosimilar pegfilgrastim would not be associated with the price of pembrolizumab, a control drug). The specific controls chosen by the model for each originator are detailed in eTable in Supplement 1. We estimated 95% prediction intervals with the estimated counterfactual ASPs to quantify prediction uncertainty.

Based on the estimated counterfactual ASPs, we produced 2 estimates. First, we estimated the relative difference in ASP, comparing the observed biologic price vs the counterfactual biologic price had biosimilars not been introduced. Second, we estimated the relative difference in the ASP, comparing the observed biosimilar price with the counterfactual originator ASP had biosimilars not been introduced. If an originator biologic had more than 1 biosimilar, we used the simple mean of available biosimilars’ ASPs to produce a single time series for effect estimation. We also repeated the analysis using the least costly biosimilar available at each time point to examine the potential ASP per dose reduction had the lowest-priced biosimilar been used instead of the corresponding originator biologic. Because the included biologics had different durations of observed postbiosimilar entry, we reported the effect estimates at 1 year, 3 years, and 5 years after biosimilar competition and as of the most recently available time point (quarter 1 of 2025).

We estimated price changes for each biologic individually and then pooled these results using a random-effects meta-analysis model. This model accounts for heterogeneity across products and weights each biologic’s effect estimate by the inverse of its variance, giving more weight to more precise estimates.

In our primary analysis, we used BSTS models to choose the control biologic drugs. In a sensitivity analysis, we also modeled counterfactual price trends using manually selected control biologics based on visual inspection for similarity of ASP trends before biosimilar market entry. We investigated the performance of BSTS with manually chosen controls (eFigure 2 in Supplement 1).

## Results

Our analysis included 7 originator biologics that entered the market between 1989 and 2004 and that had at least 3 years of postbiosimilar entry for observation (Table 1). On average, the first biosimilar for these biologics entered the market 22 years after the reference product’s market entry, with the shortest gap observed with bevacizumab (15.5 years) and the longest with epoetin (29.5 years). The number of biosimilars marketed during the study period ranged between 1 and 6 for originator biologics studied.

The ASP per dose of biologics initially decreased from their baseline value in the first 6 years (except for trastuzumab and rituximab), followed by a steady increase until the introduction of the first biosimilar. For almost all included biologics, the ASP stopped increasing and began to decrease near the first biosimilar’s market entry (eFigure 1 in Supplement 1).

**Figure 1.**
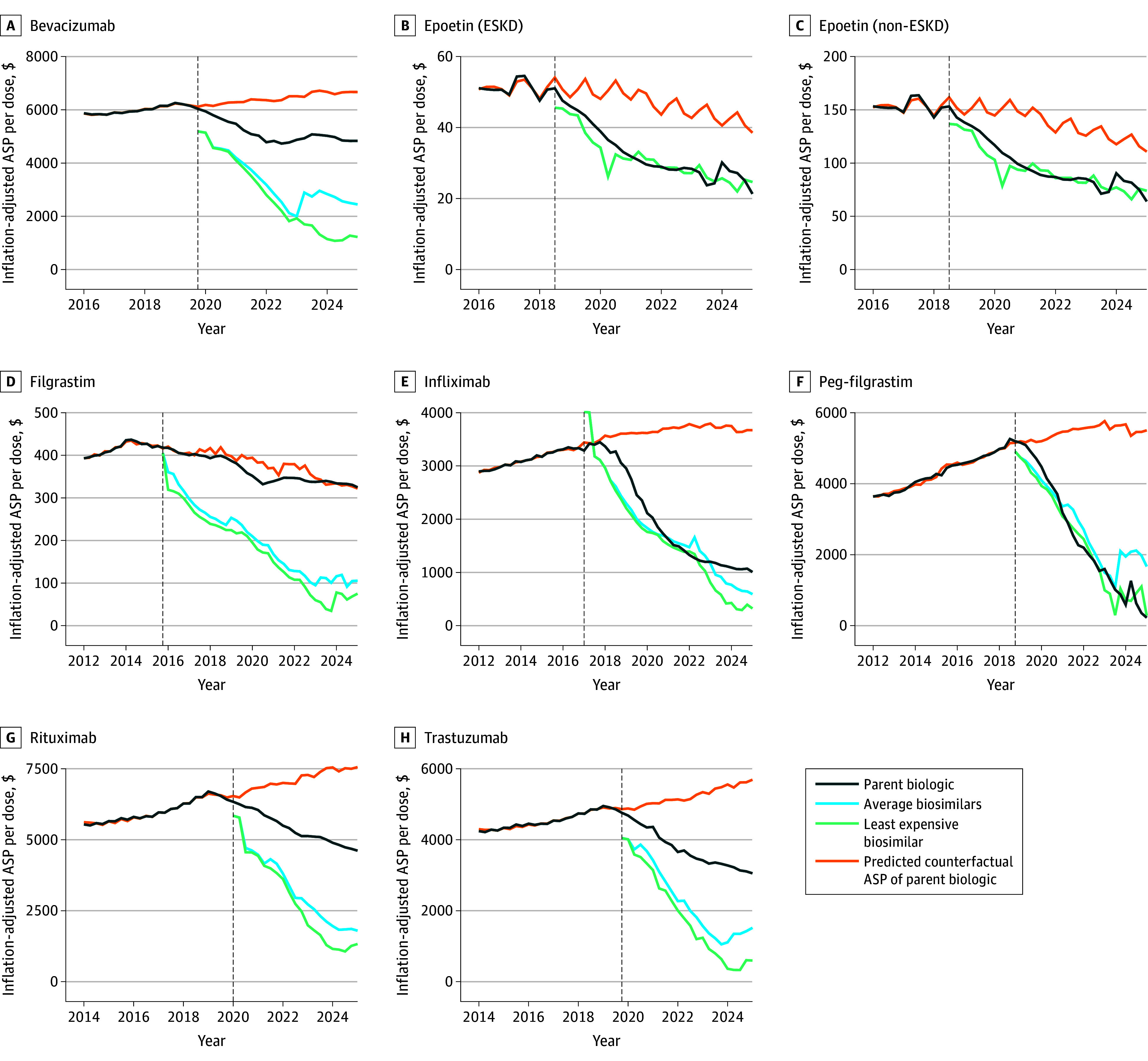
Trends in Average Sale Price (ASP) per Dose of Biologics, Average Biosimilars, and Least Expensive Biosimilar Compared With the Estimated Counterfactual ASP of Biologics The x-axis and y-axis for each panel are scaled individually to the data’s range to optimize the visualization of price trends.

Using control time series, we modeled the ASP per dose for each biologic under the counterfactual scenario of no biosimilar competition (Figure 1). Generally, our models fit the prebiosimilar trends well, shown by the overlap between the observed prebiosimilar entry and the estimated counterfactual trends. For filgrastim, epoetin (ESKD), and epoetin (non-ESKD), the amount of available control time series data was less than for other biologics, which explains the high level of uncertainty in the estimated counterfactual in the postbiosimilar entry period.

In the aggregate, we observed gradual, consistent decreases in the prices of both originator biologics and biosimilars during the first 5 years of biosimilar competition (Table 2). Relative to the estimated counterfactual biologic price, the ASP per dose of the originator biologic changed by −7.4% (95% prediction interval, –10.5% to −4.3%) at 1 year, −31.7% (95% prediction interval, –41.6% to −21.9%) at 3 years, and −43.1% (95% prediction interval, –61.3% to −24.8%), at 5 years after entry of the biosimilar competitor. Even larger price decreases were observed among the biosimilar competitors, which were priced on average −19.0% (95% prediction interval, –24.1% to −14.0%) at 1 year, −48.1% (95% prediction interval, –55.9% to −40.3%) at 3 years, and −65.3% (95% prediction interval, –74.4% to −56.3%) at 5 years relative to the price of the counterfactual biologic. Price reductions were similar when assessing prices relative to the least-costly biosimilar as opposed to mean biosimilar prices.

**Table 2.** Reduction in the ASP per Therapeutic Dose on the Introduction of Biosimilars for Each Biologic and Pooled Across all Biologics

Years after biosimilar entry	No. of available biosimilars	Change in ASP vs counterfactual of no biosimilar, %		
		Observed biologic ASP	Mean biosimilar ASP	Least costly biosimilar ASP
All drugs				
1	NA	−7.4 (−10.5 to −4.3)	−19.0 (−24.1 to −14.0)	−21.2 (−26.8 to −15.5)
3	NA	−31.7 (−41.6 to −21.9)	−48.1 (−55.9 to −40.3)	−52.2 (−61.8 to −42.7)
5	NA	−43.1 (−61.3 to −24.8)	−65.3 (−74.4 to −56.3)	−76.8 (−89.9 to −63.7)
Bevacizumab				
1	2	−8.8 (−11.9 to −5.5)	−26.9 (−30.0 to −23.7)	−27.4 (−30.4 to −24.0)
3	2	−25.6 (−30.4 to −20.3)	−60.2 (−65.0 to −54.7)	−65.4 (−70.1 to −59.7)
5	4	−27.2 (−37.3 to −14.4)	−61.5 (−71.9 to −48.8)	−83.5 (−93.7 to −70.9)
5.5	4	−27.5 (−38.5 to −13.2)	−63.3 (−74.5 to −49.1)	−81.7 (−92.8 to −67.1)
Epoetin (ESKD)				
1	1	−11.6 (−22.4 to 1.3)	−14.3 (−25.2 to −1.2)	−14.3 (−25.2 to −1.6)
3	1	−39.1 (−62.0 to −14.2)	−34.5 (−57.2 to −9.1)	−34.5 (−57.3 to −9.0)
5	1	−38.9 (−77.9 to 25.9)	−34.4 (−73.1 to 30.6)	−34.4 (−73.0 to 30.5)
6.75	1	−44.7 (−104.1 to 57.7)	−36.0 (−95.5 to 67.2)	−36.0 (−95.9 to 66.8)
Epoetin (non-ESKD)				
1	1	−11.3 (−22.3 to 3.0)	−14.1 (−25.2 to 0.3)	−14.1 (−25.2 to −0.1)
3	1	−39.2 (−63.8 to −12.2)	−34.5 (−59.2 to −7.0)	−34.5 (−59.1 to −7.4)
5	1	−37.3 (−77.9 to 30.0)	−32.7 (−74.0 to 34.0)	−32.7 (−73.3 to 34.0)
6.75	1	−42.2 (−109.0 to 68.6)	−33.2 (−101.0 to 76.2)	−33.2 (−99.7 to 75.8)
Filgrastim				
1	2	0.0 (−8.1 to 5.6)	−18.2 (−26.2 to −12.5)	−23.6 (−31.8 to −17.8)
3	2	−4.7 (−18.6 to 14.5)	−42.0 (−56.0 to −23.0)	−44.9 (−58.9 to −25.9)
5	3	−13.5 (−38.5 to 1.3)	−50.5 (−75.8 to −35.7)	−55.2 (−80.2 to −40.6)
9.5	4	0.9 (−54.6 to 55.8)	−67.3 (−122.5 to −12.4)	−76.7 (−131.5 to −21.9)
Infliximab				
1	2	−1.1 (−6.8 to 4.9)	−10.7 (−16.6 to −4.7)	−10.7 (−16.5 to −4.8)
3	2	−35.0 (−43.4 to −26.0)	−47.3 (−55.6 to −38.2)	−49.4 (−58.0 to −40.0)
5	3	−62.3 (−73.4 to −45.2)	−59.6 (−71.0 to −42.8)	−63.0 (−74.5 to −46.1)
8.25	3	−72.5 (−89.5 to −45.8)	−83.9 (−101.2 to −56.9)	−91.2 (−108.5 to −64.9)
Pegfilgrastim				
1	2	−6.6 (−14.8 to 1.8)	−14.3 (−22.9 to −5.6)	−17.6 (−25.8 to −9.0)
3	4	−54.1 (−65.4 to −34.0)	−41.0 (−52.2 to −20.8)	−50.5 (−61.7 to −30.3)
5	4	−82.0 (−95.2 to −54.0)	−81.0 (−94.4 to −53.3)	−94.8 (−108.0 to −66.3)
6.5	6	−95.9 (−109.8 to −70.1)	−69.7 (−83.7 to −42.9)	−94.9 (−108.8 to −68.4)
Rituximab				
1	2	−9.9 (−16.7 to −3.0)	−32.0 (−38.6 to −25.2)	−33.0 (−39.7 to −26.1)
3	3	−29.4 (−37.7 to −20.5)	−59.6 (−67.6 to −50.6)	−65.9 (−73.9 to −57.3)
5	3	−37.6 (−54.2 to −21.9)	−75.2 (−91.7 to −59.1)	−83.2 (−99.7 to −67.4)
5.25	3	−38.9 (−58.4 to −21.4)	−76.2 (−95.7 to −58.7)	−82.4 (−101.8 to −64.8)
Trastuzumab				
1	5	−10.0 (−15.8 to −5.0)	−21.8 (−27.6 to −16.8)	−28.9 (−34.6 to −23.8)
3	5	−30.9 (−37.4 to −24.0)	−61.2 (−67.6 to −54.7)	−69.3 (−75.7 to −62.7)
5	5	−44.1 (−56.1 to −27.7)	−76.0 (−87.8 to −59.8)	−94.1 (−106.1 to −78.3)
5.5	5	−46.3 (−59.7 to −27.9)	−73.3 (−86.9 to −54.8)	−89.5 (−103.1 to −71.1)

Abbreviations: ASP, average sale price; ESKD, end-stage kidney disease; NA, not applicable.

**Figure 2.**
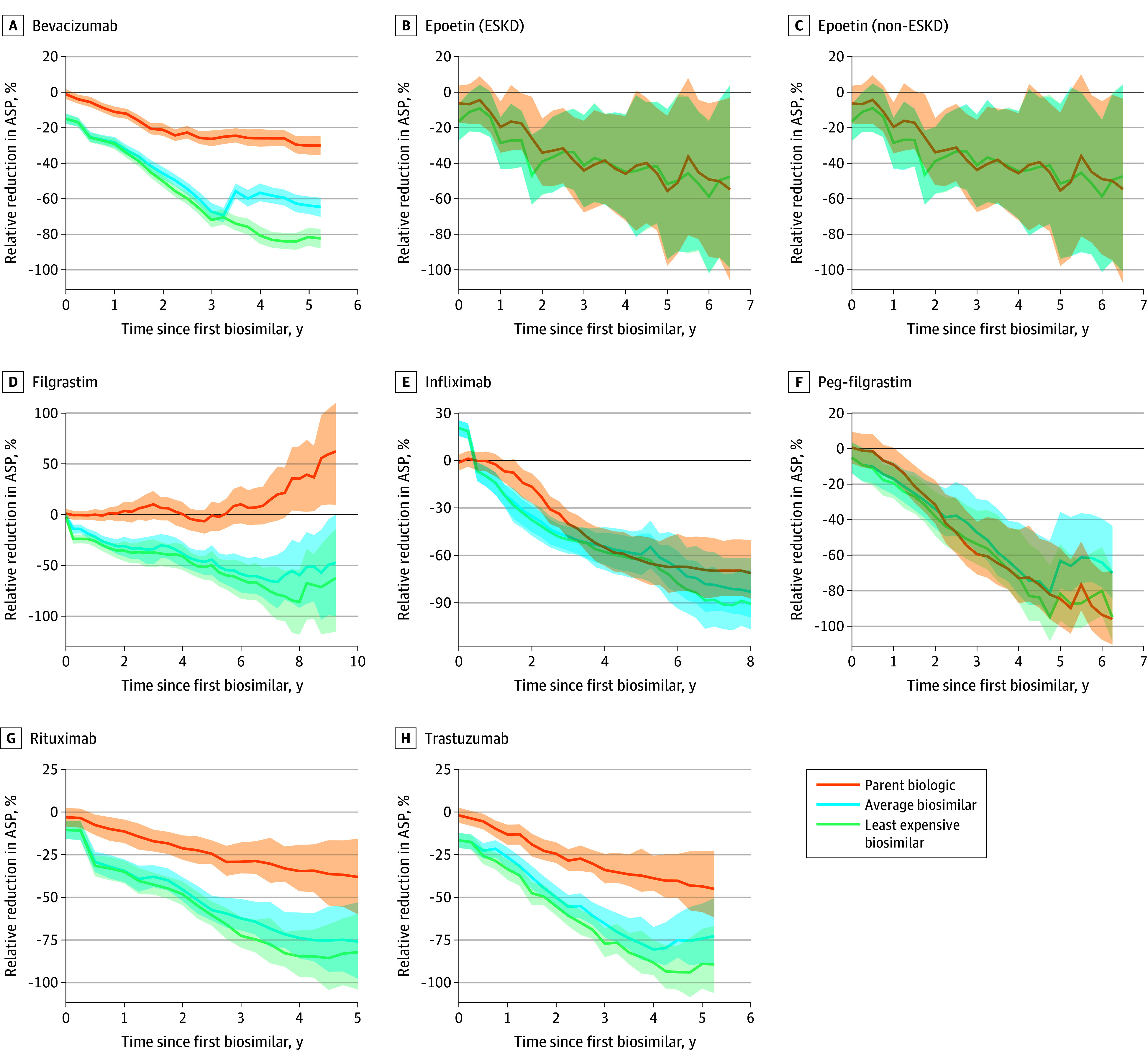
Relative Changes in Average Sale Price (ASP) per Dose (%) of the Parent Biologic, Average Biosimilar, and Least Expensive Biosimilar During the Postbiosimilar Period, Both vs the Counterfactual Parent Biologic ASP in the Absence of Biosimilar Competition The x-axis and y-axis for each panel are scaled individually to the data’s range to optimize the visualization of price trends. The biosimilars line reflects the ASP of all available biosimilars at each time point. The shaded areas represent the 95% prediction intervals.

In general, the prices of the individual drug groups followed these overall trends (Table 2 and Figure 2). Savings varied by drug, with the largest price reductions observed with pegfilgrastim and infliximab, with 5-year price reductions of −82.0% (95% prediction interval, –95.2% to −54.0%) for pegfilgrastim and −62.3% (95% prediction interval, –73.4% to −45.2%) for infliximab. Biosimilars generally achieved greater reductions in the ASP per dose compared with originator biologics. Rituximab and trastuzumab were the only biologics with a significant reduction in their ASP per dose within 1 year after the entry of biosimilars. Pegfilgrastim was the originator biologic with the greatest reduction in the ASP per dose compared with the corresponding biosimilars after 1 year of competition. Three years after biosimilar entry, pegfilgrastim had the greatest reduction in ASP per dose (−54.1% [95% prediction interval, –65.4% to −34.0%]). The most pronounced divergence between originator biologics and biosimilars was observed with filgrastim, wherein the originator biologic’s ASP per dose remained largely unchanged (0.9% [95% prediction interval, –54.6% to 55.8%]), despite substantial price decreases by its biosimilar competitors (−67.3% [95% prediction interval, –122.5% to −12.4%]) (Figure 2). Except for filgrastim, the ASP per dose for all biologics showed a decreasing trend in the subsequent years. For some biologics, namely bevacizumab, rituximab, and trastuzumab, the gap in the reduction of ASP per dose by using the originator biologic remained steady over time (Figure 2). However, for other biosimilars (epoetin, infliximab, and pegfilgrastim), the price differential between biologic and biosimilar decreased over time, resulting in similar reductions in the ASP per dose (Table 2 and Figure 2). Average sale price per dose reductions were similar for epoetin (ESKD), epoetin (non-ESKD), infliximab, and pegfilgrastim and were slightly greater for bevacizumab, filgrastim, trastuzumab, and rituximab (Table 2 and Figure 2).

## Discussion

In this cohort study, we estimated the association of biosimilar entry with originator biologics’ prices in Medicare Part B relative to a counterfactual setting in which biosimilar entry did not occur. By comparing with a counterfactual in which the originator biologic price was likely to continue increasing in the absence of biosimilar competition, our approach may more accurately capture the full associations of biosimilars with originator biologics’ prices relative to prior studies. Overall, we found that biosimilar entry was associated with reduced prices of both the originator biologic and biosimilar competitor over time (Table 2).

Previous studies have typically found that biosimilar competition results in modest reductions in biologic drug prices. For instance, compared with estimates from the US Department of Health and Human Services Office of Inspector General’s (OIG) report,^20^ which compared observed prices after biosimilar entry with prices immediately before biosimilar availability, we found larger price reductions for bevacizumab (−25.6% in the present study vs −13% in the OIG report), rituximab (−9.9% vs −8%), and trastuzumab (−30.9% vs −16%) in similar postbiosimilar entry periods. Similarly, a 2025 report from the Office of the Assistant Secretary for Planning and Evaluation also examined these price dynamics, finding modest price reductions after biosimilar entry.^33^ These differences likely arise from our purposefully different approach of comparing postcompetition prices with a counterfactual price rather than simply with the historical precompetition price.^19,20^ In addition, our results underscore the heterogeneity of associations of biosimilar competition with prices of biologics across different biologics, which aligns with prior research showing variations in price decreases due to factors such as manufacturing complexities, regulatory challenges, and competitive strategies by originator companies.^6,8,10,34^ Previous research has noted that most biologic manufacturers reduce prices to retain market share in the face of biosimilar competition.^34^ However, in some cases (notably, filgrastim), the manufacturer’s strategy was to maintain the originator price and accept a lower market share.^34^ Our results extend this prior work by documenting the stability of these trends over a more extended postbiosimilar entry period.

We also considered whether the number of competitors correlated with the magnitude of price reduction given the same time frame. Although our small sample size precludes a formal analysis of this association, we did not observe a clear pattern. For instance, at the 3-year mark, pegfilgrastim saw the largest price reduction, with 4 biosimilars on the market. In contrast, trastuzumab saw a smaller price reduction despite having 5 biosimilars. Meanwhile, epoetin experienced substantial price decreases of more than 40%, with only 1 biosimilar competitor at the 3-year mark.

Our results demonstrate substantial originator product price reductions after biosimilar entry (ranging from approximately 0% to 11.6% within 12 months after biosimilars’ approval). Even after 5 years of biosimilar competition, these price reductions are smaller than those observed with small-molecule generic drugs, where price reductions are typically 75% or more within the first year.^35^ The more modest price decreases for biosimilars may be associated with several factors, including higher development and manufacturing costs, more complex regulatory requirements, smaller numbers of competitors compared with the generic small-molecule market, and lower uptake of biosimilars among health plans, health care organizations, and patients.^1,17,18,36^ The relatively small price difference between some originator biologics and their biosimilar counterparts has important implications for the market for biosimilars. For example, the smaller price differentials between biosimilars and originator biologics may reduce the incentive for payers and clinicians to switch to biosimilars, potentially limiting their market penetration and overall cost-saving potential. Recognizing this incentive problem, policymakers included a provision in the Inflation Reduction Act of 2022 to temporarily increase the add-on payment for qualifying biosimilars from 6% to 8% of the originator’s ASP in an effort to encourage their adoption.^29^ In the short run, this may reflect rational payer and clinician behavior. However, in the long run, these choices may disincentivize biosimilar development, allowing manufacturers of originator biologics to maintain high prices without a threat of biosimilar competition.

Although our study shows that biosimilar entry is associated with substantial price reductions, their full cost-saving potential depends on widespread adoption. Future research should include market volume and adoption rates to better estimate actual cost savings. Our findings also suggest that policymakers may need to consider additional incentives to promote biosimilar use because the price decreases for biologics remain more modest than those for generic drugs.

### Limitations

Our analysis has several limitations. First, for some control drugs, the data were insufficient to generate a reliable counterfactual, leading to greater uncertainty in our price estimations, particularly for drugs such as epoetin. The selection of controls, while deliberate, may not fully capture all market dynamics unique to specific drugs or therapeutic areas. In addition, the number and predictive quality of controls varied among the biologics in our sample, affecting the precision of some counterfactual estimates. Second, our findings are specific to the Medicare Part B context and may not apply to other payers or health care systems. Third, there is a 2- to 3-quarter lag before CMS can calculate a biosimilar’s ASP. During this time, payment is based on the Wholesale Acquisition Cost. The public data files do not distinguish between Wholesale Acquisition Cost– and ASP-based payments, making our calculated ASPs for a biosimilar’s first few quarters less precise. Therefore, our findings for the first year of competition should be interpreted with caution.^37^

## Conclusion

Our findings in this cohort study suggest that price decreases for biologic drugs may have been greater than previously estimated.^19^ Nevertheless, the price reductions achieved by biosimilar competition remain modest when benchmarked against the savings observed with generic small-molecule drugs. The associations of biosimilar entry with prices of originator biologics varied across biologics, with most biologics exhibiting a gradual decrease in ASP after the introduction of the first biosimilar competitor. Biosimilars were generally priced below their reference biologics. These findings suggest that while biosimilars have the potential for cost savings, additional measures are needed to fully realize their effect on Medicare Part B spending. Future research should explore biosimilar adoption factors and market dynamics to better understand their economic potential.

## Notes

Supplement 1. **eTable.** Selected Controls for Bayesian Structural Time Series Modeling **eFigure 1.** Average Sale Price (ASP) per Dose of Biologic Drugs With Biosimilar Competitors by 2021 **eFigure 2.** Comparing the Performance of Models That Included all Controls (Left Panel) Versus Models That Included Manually Selected Controls (Right Panel) Concerning Trends in Average Sale Price (ASP) per Dose of Biologics (Black) Compared to the Predicted Counterfactual Trends of Biologics (Dashed Blue)

Supplement 2.
Data Sharing Statement
