# Supplementary material for: Biologic Drug Prices in Medicare Part B After Entry of Biosimilars to the Market
Source: JAMA Netw Open. 2025 Nov 11;8(11):e2542937. doi: 10.1001/jamanetworkopen.2025.42937 (PMC12606378; doi:10.1001/jamanetworkopen.2025.42937)
Supplement: Supplement 1. — eTable. Selected Controls for Bayesian Structural Time Series Modeling eFigure 1. Average Sale Price (ASP) per Dose of Biologic Drugs With Biosimilar Competitors by 2021 eFigure 2. Comparing the Performance of Models That Included all Controls (Left Panel) Versus Models That Included Manually Selected Controls (Right Panel) Concerning Trends in Average Sale Price (ASP) per Dose of Biologics (Black) Compared to the Predicted Counterfactual Trends of Biologics (Dashed Blue) [file jamanetwopen-e2542937-s001.pdf]

Supplementary Online Content

Abdelaziz A, Winn AN, Dusetzina SB, Mitchell AP. Biologic drug prices in Medicare Part B after entry of biosimilars to the market. *JAMA Netw Open.* 2025;8(11):e2542937. doi:10.1001/jamanetworkopen.2025.42937

eTable. Selected Controls for Bayesian Structural Time Series Modeling

eFigure 1. Average Sale Price (ASP) per Dose of Biologic Drugs With Biosimilar Competitors by 2021

eFigure 2. Comparing the Performance of Models That Included all Controls (Left Panel) Versus Models That Included Manually Selected Controls (Right Panel) Concerning Trends in Average Sale Price (ASP) per Dose of Biologics (Black) Compared to the Predicted Counterfactual Trends of Biologics (Dashed Blue)

This supplementary material has been provided by the authors to give readers additional information about their work.

© 2025 Abdelaziz A et al. *JAMA Network Open.*

eTable. Selected Controls for Bayesian Structural Time Series Modeling

Drug	Manually-chosen controls	All potential controls
**Bevacizumab**	Denosumab, Nivolumab, Pembrolizumab, Pertuzumab, Ustekinumab (SC)	Abatacept, Aflibercept, Denosumab, Nivolumab, Pembrolizumab, Pertuzumab, Pneumococcal vaccine (13-valent), Pneumococcal vaccine, Tocilizumab, Ustekinumab (SC), ziv-Aflibercept
**Epoetin (ESRD)**	Denosumab, Nivolumab, Pembrolizumab, Pertuzumab, Ustekinumab (SC), ziv-Aflibercept	Abatacept, Aflibercept, Denosumab, Nivolumab, Pembrolizumab, Pertuzumab, Pneumococcal vaccine (13-valent), Pneumococcal vaccine, Tocilizumab, Ustekinumab (SC), ziv-Aflibercept
**Epoetin (non-ESRD)**	Denosumab, Nivolumab, Pembrolizumab, Pertuzumab, Ustekinumab (SC), ziv-Aflibercept	Abatacept, Aflibercept, Denosumab, Nivolumab, Pembrolizumab, Pertuzumab, Pneumococcal vaccine (13-valent), Pneumococcal vaccine, Tocilizumab, Ustekinumab (SC), ziv-Aflibercept
**Filgrastim**	Denosumab, Pneumococcal vaccine (13-valent), Ustekinumab (SC)	Abatacept, Denosumab, Pneumococcal vaccine (13-valent), Pneumococcal vaccine, Tocilizumab, Ustekinumab (SC)
**Infliximab**	Denosumab, Tocilizumab, Pneumococcal vaccine (13-valent), Ustekinumab (SC)	Abatacept, Denosumab, Pneumococcal vaccine (13-valent), Pneumococcal vaccine, Tocilizumab, Ustekinumab (SC)
**Peg-filgrastim**	Denosumab, Tocilizumab, Pneumococcal vaccine	Abatacept, Denosumab, Pneumococcal vaccine (13-valent), Pneumococcal

© 2025 Abdelaziz A et al. *JAMA Network Open.*

	(13-valent), Ustekinumab (SC)	vaccine, Tocilizumab, Ustekinumab (SC)
**Rituximab**	Denosumab, Tocilizumab, Pertuzumab, Ustekinumab (SC)	Abatacept, Aflibercept, Denosumab, Pertuzumab, Pneumococcal vaccine (13-valent), Pneumococcal vaccine, Tocilizumab, Ustekinumab (SC), ziv-Aflibercept
**Trastuzumab**	Denosumab, Pertuzumab, Tocilizumab, Ustekinumab (SC)	Abatacept, Aflibercept, Denosumab, Pertuzumab, Pneumococcal vaccine (13-valent), Pneumococcal vaccine, Tocilizumab, Ustekinumab (SC), ziv-Aflibercept

Abbreviations ESRD: End-Stage Renal Disease; SC: Subcutaneous

© 2025 Abdelaziz A et al. *JAMA Network Open.*

eFigure 1. Average Sale Price (ASP) per Dose of Biologic Drugs With Biosimilar Competitors by 2021. Prices are inflated to Q1 2025 U.S. dollars. The vertical dashed line represents the first biosimilar entry, and the horizontal dotted line represents the baseline price in Q1 2005.

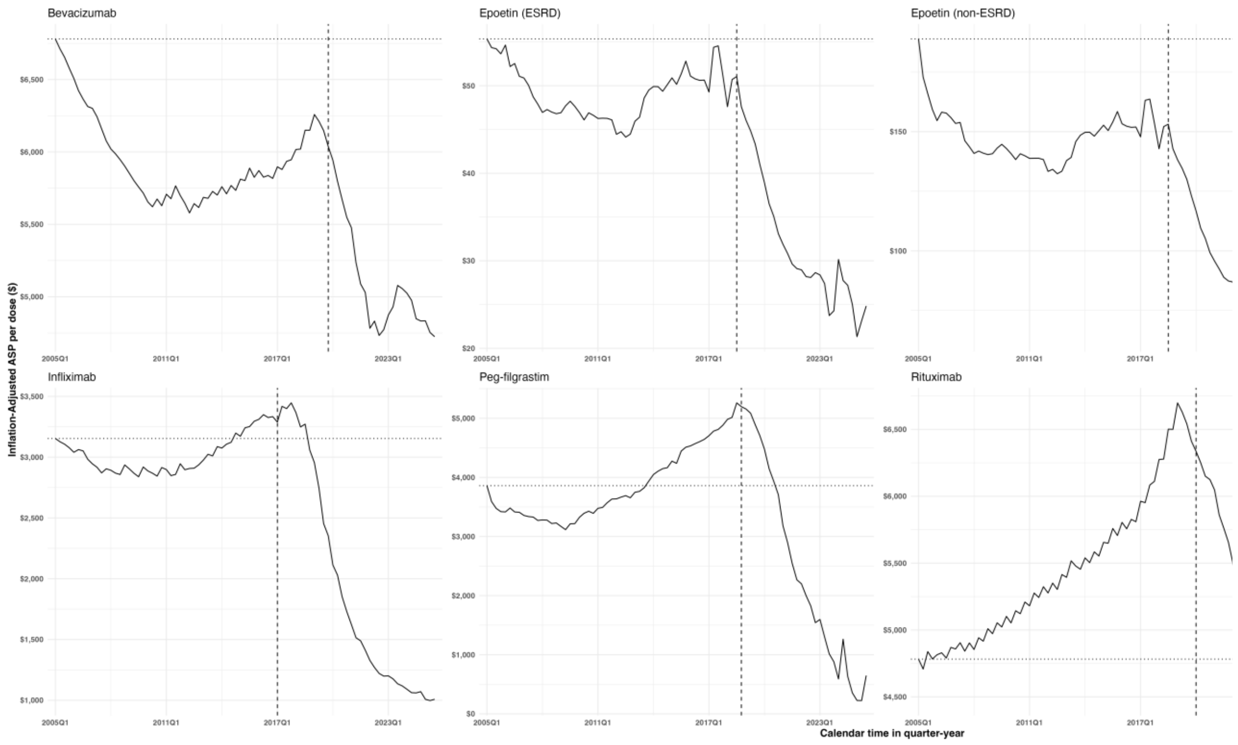

The x-axis and y-axis for each panel are scaled individually to the data's range to optimize the visualization of price trends.

eFigure 2. Comparing the Performance of Models That Included all Controls (Left Panel) Versus Models That Included Manually Selected Controls (Right Panel) Concerning Trends in Average Sale Price (ASP) per Dose of Biologics (Black) Compared to the Predicted Counterfactual Trends of Biologics (Dashed Blue). The red ribbon represents the 95% prediction intervals.

© 2025 Abdelaziz A et al. *JAMA Network Open.*

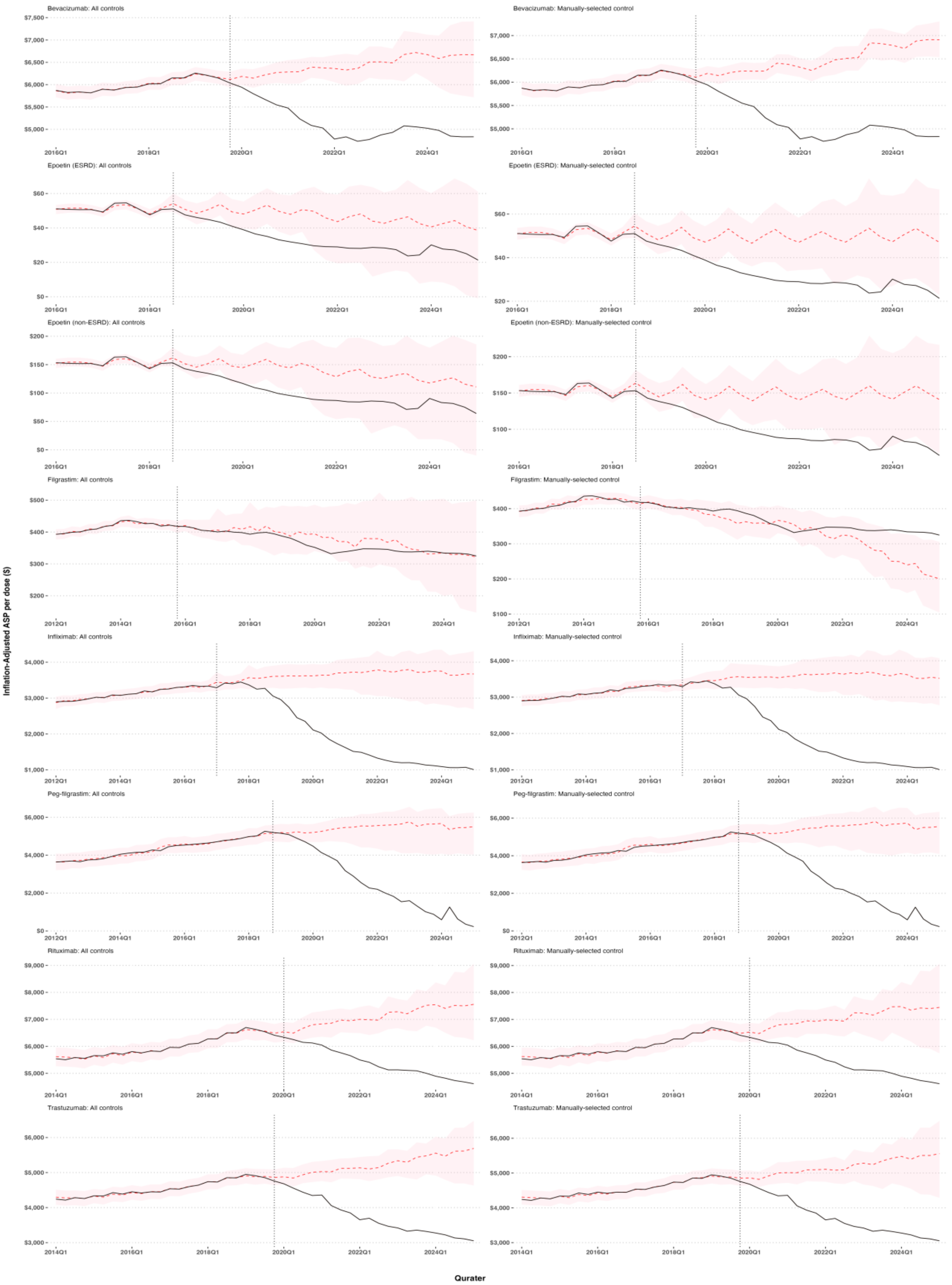

© 2025 Abdelaziz A et al. *JAMA Network Open.*
