# Supplementary material for: Biologic Drug Prices in Medicare Part B After Entry of Biosimilars to the Market
Source: JAMA Netw Open. 2025 Nov 11;8(11):e2542937. doi: 10.1001/jamanetworkopen.2025.42937 (PMC12606378; doi:10.1001/jamanetworkopen.2025.42937)
Supplement: Supplement 2. — Data Sharing Statement [file jamanetwopen-e2542937-s002.pdf]

Data Sharing Statement

Abdelaziz. Biologic Drug Prices in Medicare Part B After Entry of Biosimilars to the Market. *JAMA Netw Open*. Published November 11, 2025. doi:10.1001/jamanetworkopen.2025.42937

Data
Data available: No

Additional Information
Explanation for why data not available: This study used Average Sales Price data, which is available for free public download from the Centers for Medicare and Medicaid Services (CMS)
